# Choledochal Cyst and Right Congenital Diaphragmatic Hernia: When to Intervene?

**DOI:** 10.1055/s-0044-1791813

**Published:** 2024-10-28

**Authors:** Émilie Kate Landry, Annie Le-Nguyen, Elissa K. Butler, Sarah Bouchard, Josée Dubois, Caroline P. Lemoine

**Affiliations:** 1 Division of Pediatric Surgery, CHU Sainte-Justine, Montreal, Quebec, Canada; 2 Department of Radiology, Radiation Oncology, and Nuclear Medicine, CHU Sainte-Justine, Montreal, Quebec, Canada

**Keywords:** choledochal cyst, biliary complications, congenital diaphragmatic hernia, mesh repair

## Abstract

Patients with congenital diaphragmatic hernia (CDH) can present with other congenital anomalies, but an associated choledochal cyst (CC) has rarely been described. The simultaneous diagnosis of both anomalies complicates patient management. There is no consensus on the ideal timing for CC excision. Unrepaired CC is associated with risks of developing biliary sludge, choledocholithiasis, and cholangitis. After a CDH repair with mesh, secondary bacterial translocation caused by a delayed CC repair could lead to mesh superinfection. Conversely, early CC surgical management could cause mesh displacement and lead to CDH recurrence, requiring reintervention. We present the rare case of a CC occurring in a neonate with a prenatally diagnosed right CDH. One month after an uncomplicated CDH repair with mesh, while the patient was still hospitalized for pulmonary hypertension, she developed progressive cholestasis and acholic stools. Investigations revealed a nonpreviously suspected CC. Conservative treatment was attempted, but CC perforation with secondary biliary peritonitis occurred. Open CC excision with a Roux-en-Y hepaticojejunostomy was therefore performed on day of life (DOL) 41. Having suffered no short-term surgical complications, the patient was discharged on DOL 83 because of prolonged ventilatory support due to pulmonary hypertension. Now 12 months after surgery, she is doing well with normal liver function tests and imaging studies. In summary, CC should be considered in the differential diagnosis of progressive cholestasis in patients with CDH. Surgical repair of a symptomatic CC should not be delayed even in the presence of mesh given the risks of CC complications.

## Introduction

Congenital diaphragmatic hernia (CDH) is a rare anomaly that results in herniation of abdominal content into the chest. Most CDH involve a left posterolateral defect, and the reported incidence of right-sided CDH is only 10 to 15%. 1 The overall incidence of CDH is approximately 1 in 2,500 to 5,000 live births. 2 It is often prenatally diagnosed and is associated with long-term morbidity and a high mortality rate due to respiratory distress at birth. 3 While smaller diaphragmatic defects may be closed primarily, larger defects require either muscle flap or mesh closure. 4

CDH is associated with other congenital malformations in 40% of cases. 5 Cardiac, tracheobronchial, neurologic, and limb malformations are commonly found. 3 However, the simultaneous occurrence of a CDH and a choledochal cyst (CC) has only been reported once in the literature (left-sided CDH primarily repaired). 6 CC consists of a congenital biliary dilatation and is a rare entity in Western countries (1:100,000–150,000 births), but is more commonly found in Asia (1:1,000 births). 7 It is often diagnosed incidentally on imaging performed for other indications. 8 When symptomatic, neonates may present with obstructive jaundice, acholic stool, or hepatomegaly. In older children, abdominal pain is the most frequent presentation of CC. However, the classic triad of abdominal pain, jaundice, and a palpable mass is uncommon. 8 Surgical excision is recommended due to the risks of complications such as cholangitis and pancreatitis but also malignant transformation. Optimal timing for surgical management is still debated. 9 10 11

We present the first reported case of a female newborn with a prenatally diagnosed right CDH and, in hindsight, a missed CC. The objective of this report is to discuss the complex decision-making involved in the management of both anomalies when they occur simultaneously, as well as the risks and benefits of early versus delayed CC excision with the added complexity of a CDH mesh closure.

## Case Report

A female baby was diagnosed with a right CDH on prenatal ultrasound (US) late during pregnancy at 29 weeks of gestational age (GA; the pregnancy was only discovered during the second trimester). The mother's four previous pregnancies were uneventful and the family history was unremarkable. The first prenatal US was performed at 27 weeks of GA, which revealed a right thoracic mass causing a left mediastinal shift. At 29 weeks of GA, polyhydramnios and a right CDH were diagnosed. The stomach and liver were located intra-abdominally and only bowel loops were herniated into the chest. The lung-to-head ratio was 1.9. No fetal magnetic resonance imaging (MRI) was obtained. An amniocentesis was found to be normal. In light of an advanced pregnancy, genetic paneling was deferred postnatally.

At 38 weeks of GA, an emergency cesarean section was performed because of uterine bleeding and fetal transverse presentation. The newborn girl was rapidly intubated after birth because of respiratory distress and an Apgar score of 1-3-3, and then admitted to the neonatal intensive care unit. An echocardiogram showed normal heart function and no pulmonary hypertension. As the patient was on minimal ventilator settings and cardiovascular support, CDH surgical repair was performed on day of life (DOL) 3 through a right subcostal incision. Once the bowel and liver were reduced, a right hypoplastic lung and a large defect with only a small anterior diaphragmatic rim were identified. As primary closure was impossible, a polytetrafluoroethylene mesh was anchored posteriorly to the rib cage using 2–0 Ethibond and pledgets. There was no immediate complication, but pulmonary hypertension was noted postoperatively. Treatment with nitric oxide was initiated and sildenafil was eventually started. She was extubated on postoperative day 13 (DOL 16) and placed on continuous positive airway pressure (CPAP) therapy until DOL 37. Enteral feeds were started on postoperative day 14 (DOL 17) and total parenteral nutrition (TPN) was stopped 3 days later (DOL 20).

**Fig. 1:**
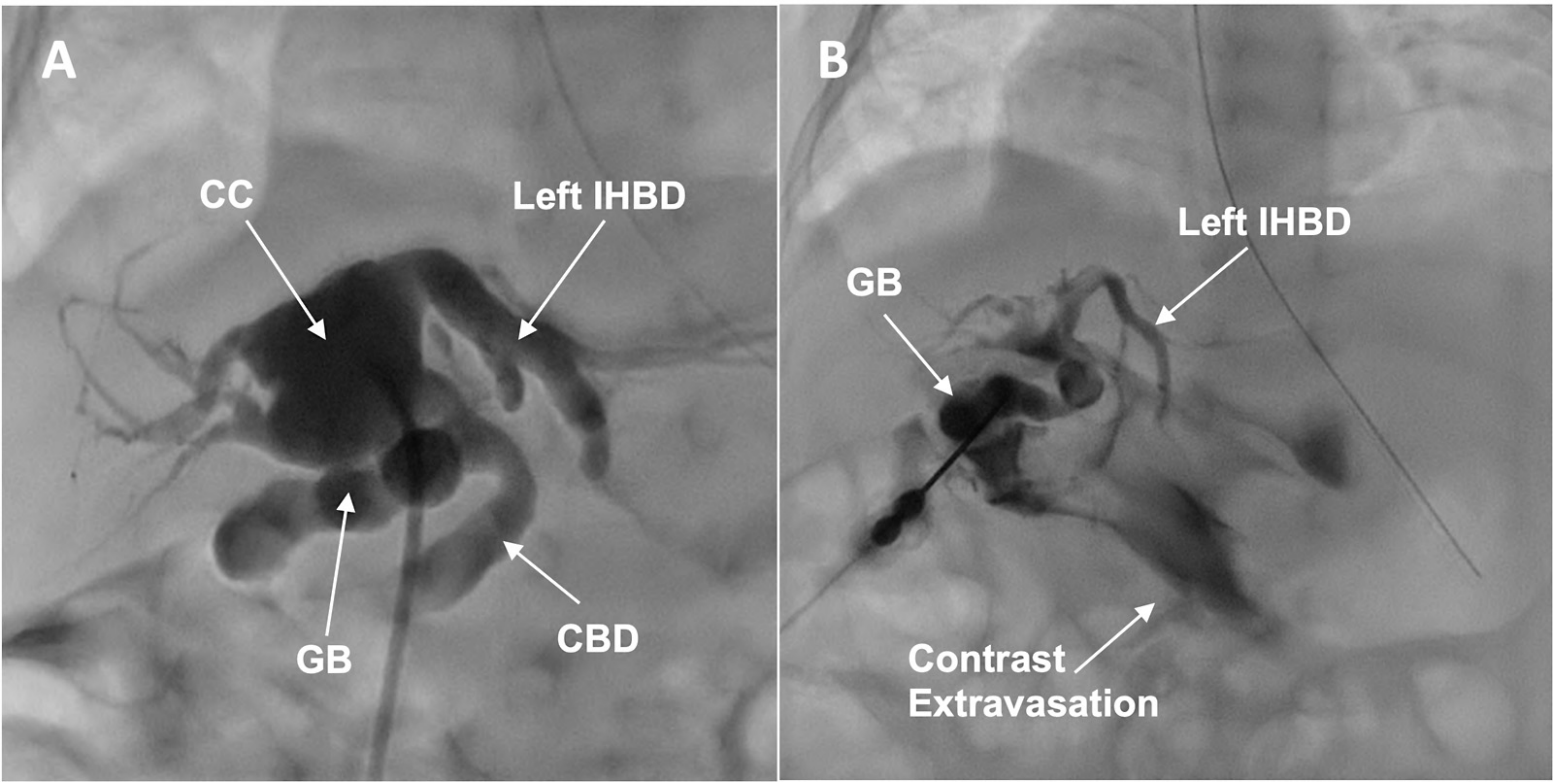
Percutaneous transhepatic cholangiography (PTC) results. ( **A** ) First PTC: after clearing sludge, an abnormally dilated biliary tree compatible with a CC was unexpectedly found. ( **B** ) Second PTC: contrast extravasation in the peritoneal cavity is seen, compatible with CC rupture. CC, choledochal cyst; CBD, common bile duct; GB, gallbladder; IHBD, intrahepatic bile duct.

**Fig. 2:**
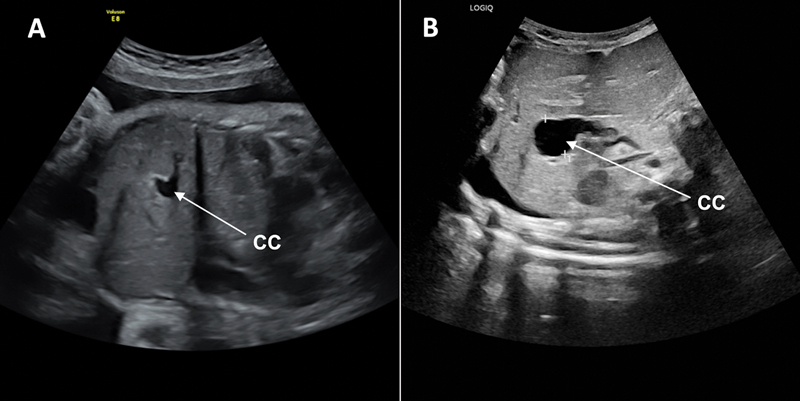
Ultrasound (US) imaging showing the intrahepatic biliary structure originally identified as the gallbladder, but ultimately found to be the choledochal cyst (CC). ( **A** ) Prenatal US done at 34 weeks of gestational age. ( **B** ) US performed at 32 days of life when investigating cholestasis.

On DOL 31, while she was tolerating full enteral feeds, she developed cholestasis (total/direct bilirubin: 3.6/2.8 mg/dL; gamma-glutamyl transferase [GGT]: 1050 U/L) and acholic stools. An US showed biliary dilatation and sludge. The following day, a percutaneous transhepatic cholangiography (PTC) successfully cleared the sludge but at the same time revealed a type I CC involving the common hepatic channel ( Fig. 1A ). Given this new diagnosis, a magnetic resonance cholangiopancreatography (MRCP) was obtained after the PTC, showing a persistently dilated right common hepatic duct, albeit less dilated than previously described. However, the previously dilated central common hepatic channel could no longer be visualized as the sludge causing obstruction had been cleared. Pre- and postnatal imaging was reviewed and confirmed that the CC could be seen but was misinterpreted as the gallbladder ( Fig. 2 ). Multidisciplinary discussions were held focusing on the optimal timing for CC surgical repair. The plan was to delay surgery to allow for pulmonary hypertension to improve or resolve, and also to decrease risks of mesh displacement and/or superinfection. Broad-spectrum antibiotics and ursodeoxycholic acid were started, but despite this, liver function tests further deteriorated 8 days later (total/direct bilirubin: 6.6/4.7 mg/dL). An US was obtained, which showed decompressed intrahepatic bile ducts and new free fluid. A repeat PTC confirmed CC perforation with biliary peritonitis ( Fig. 1B ). On DOL 41, the patient was taken to the operative room for the definitive surgical management of the CC. A new right subcostal incision lower than the previous one used for the CDH repair was performed. On entering the abdominal cavity, a large amount of bile was aspirated and sent for culture. The liver was carefully retracted to expose the porta hepatis. The mesh was not visualized. As tissues were inflamed, intraoperative US guidance was used to distinguish the common bile duct (CBD) from adjacent vascular structures. The perforation site could be seen anteriorly close to the insertion of the cystic duct. The CC was excised until reaching the bifurcation of the right and left intrahepatic bile ducts. They were left on a common patch. A cholecystectomy was performed at the same time. Then, an end-to-side jejunojejunostomy was performed, a 45-cm Roux limb was created, passed in a retrocolic fashion, and an end-to-side hepaticojejunostomy was done using 5–0 monofilament running sutures. A closed-suction drain was placed posterior to the anastomosis.

The patient did well from a CC standpoint. There was no immediate complication. A follow-up US was obtained 1 month postoperatively while the patient was still admitted. It did not show any anomaly (no intrahepatic bile duct dilatation) and liver function tests were improved (total/direct bilirubin: 0.7/0.4 mg/dL; GGT: 280 U/L). She was discharged on sildenafil 42 days after CC surgery (DOL 83) without any oxygen or ventilatory support. Now 12 months after discharge, the patient is followed every 3 months by our institutional CDH multidisciplinary clinic with liver function tests, a liver US, and pulmonary imaging. She is doing well: pulmonary hypertension has resolved, liver function tests have normalized (total/direct bilirubin: 0.3/0.1 mg/dL; GGT 24 U/L), and imaging studies show no biliary dilatation or CDH recurrence. She is eating normally by mouth and does not suffer from gastroesophageal reflux. She does not exhibit any evidence of scoliosis. She is also, overall, developing well from a neurodevelopmental standpoint as she is able to stand up by herself.

## Discussion

This is the second reported case of concomitant CC and CDH in a newborn but the first report of a CC and a right CDH repaired with a mesh.

There is no known embryogenic hypothesis to explain the simultaneous occurrence of both anomalies. Environmental factors, abnormal molecular pathways, genetic syndromes, and chromosomal anomalies are actively being studied as potential CDH etiologies. 3 12 A recent study has postulated that oxidative stress during the early stages of embryogenesis may be responsible for CDH as well as for congenital heart defects, biliary atresia, esophageal atresia, and dominant polycystic kidney disease. 13 This may explain why newborns with CDH are 10 to 50% more likely to have associated anomalies than the general population. 6 For CC, 90% of cases appear to be related to anomalies of the pancreaticobiliary junction. 7 The embryological development of the diaphragm, pancreas, and bile ducts all occur between the fourth and eighth gestational weeks. 14 15 It is therefore plausible that an in utero event occurring at that time could impact all those organs and lead to the concomitant development of both congenital anomalies.

Physicians must consider congenital conditions when new symptoms arise in patients with CDH. In this case, a CC was not originally suspected since cholestasis could be explained by perioperative hemodynamic instability after the CDH repair, CDH cardiovascular physiology, and TPN administration. Retrospective evaluation of the patient's imaging confirmed that the anomaly was present prenatally but likely overlooked in the setting of another major congenital anomaly. Prenatal MRI could have potentially helped better characterize the biliary tree anatomy.

Open excision with a Roux-en-Y hepaticojejunostomy remains the gold standard for CC surgical treatment, but there is growing evidence that a laparoscopic intervention yields similar results. 9 However, when managing a perforated CC (incidence of 1.8–12.2%), 16 laparotomy is the preferred approach given the inflammation found in the porta hepatis that can further lead to portal vein thrombosis and portal hypertension. While there is consensus in performing surgery for symptomatic patients with CC to avoid developing further complications, the optimal surgical repair timing for an asymptomatic patient with a prenatally diagnosed CC remains uncertain. In Asia, surgery is commonly performed in the first 2 months of life for prenatally diagnosed CC. 9 Although in North America or Europe surgery is typically postponed after 6 months of age, 17 a recent single-center retrospective study suggests resection is safe and feasible in neonates without a higher complication rate. 10

Primary closure is the preferred CDH surgical management, but when a large defect is identified, a muscle flap or mesh closure is required. 4 18 CDH recurrence is a feared short-term complication after surgical repair, especially when a mesh is used. 19 In this presented case, due to the absence of significant diaphragmatic rim, the mesh was anchored directly to the posterolateral chest wall, providing less strength. Given a high risk of recurrence, early CC excision was initially not favored to avoid possible mesh displacement at the time of a redo right subcostal laparotomy. Secondary infections also constitute an early mesh complication, which may require mesh removal to provide definitive infectious treatment. 20 In our patient's case, the CC posed risks of cholangitis with bacterial translocation potentially leading to secondary mesh superinfection. Although there is no clear definition of a CDH mesh superinfection, suspected or confirmed cases are highly morbid and potentially fatal, and can even occur several years after surgery. 20 Therefore, because of the potential risks of mesh contamination from biliary spillage at the time of CC repair, initial conservative management with ursodeoxycholic acid, prophylactic antibiotics, and biliary sludge clearance by PTC was attempted. However, when CC rupture with biliary peritonitis occurred, surgery became necessary. In hindsight, although the patient did not develop any infectious complications from biliary peritonitis, early surgical management with a more controlled bile contamination could have represented an overall lower mesh infectious risk.

In conclusion, this is the first reported case depicting the simultaneous occurrence of a CC and a right CDH. It highlights how new symptoms occurring in patients with CDH should raise suspicion of another congenital anomaly as part of the differential diagnosis. Based on our experience, we recommend early surgical management of any symptomatic CC. It should not be delayed despite a previous history of CDH repair with mesh as the risks of CC complications, such as perforation and infection, and their potential impact on mesh complications outweigh those of CC surgery.

## Notes

Importance for a Pediatric Surgeon Choledochal cyst must be included in the differential diagnosis of cholestasis in a neonate with a repaired congenital diaphragmatic hernia. Early choledochal cyst excision is recommended even shortly after diaphragmatic hernia repair with mesh as the complication risks from the cyst itself outweigh those of surgery.
